# What happens in the brain when we die? Deciphering the neurophysiology of the final moments in life

**DOI:** 10.3389/fnagi.2023.1143848

**Published:** 2023-05-09

**Authors:** Nathan A. Shlobin, Jaan Aru, Raul Vicente, Ajmal Zemmar

**Affiliations:** ^1^Department of Neurosurgery, Henan Provincial People’s Hospital, Henan University School of Medicine, Zhengzhou, China; ^2^Department of Neurological Surgery, Northwestern University Feinberg School of Medicine, Chicago, IL, United States; ^3^Institute of Computer Science, University of Tartu, Tartu, Estonia; ^4^Department of Neurological Surgery, University of Louisville School of Medicine, Louisville, KY, United States

**Keywords:** dying brain, death, near-death experiences, oscillations, memory recall, consciousness

## Abstract

When do we die and what happens in the brain when we die? The mystery around these questions has engaged mankind for centuries. Despite the challenges to obtain recordings of the dying brain, recent studies have contributed to better understand the processes occurring during the last moments of life. In this review, we summarize the literature on neurophysiological changes around the time of death. Perhaps the only subjective description of death stems from survivors of near-death experiences (NDEs). Hallmarks of NDEs include memory recall, out-of-body experiences, dreaming, and meditative states. We survey the evidence investigating neurophysiological changes of these experiences in healthy subjects and attempt to incorporate this knowledge into the existing literature investigating the dying brain to provide valuations for the neurophysiological footprint and timeline of death. We aim to identify reasons explaining the variations of data between studies investigating this field and provide suggestions to standardize research and reduce data variability.

## Introduction

The age-old question of what happens in our brain when we die has engaged mankind for centuries. Perhaps the only possibility to receive answers to this question are the subjective descriptions of individuals who came close to death but survived, so-called near-death survivors. These near-death experiences (NDEs) were described in Moody (1975), reporting the descriptions of hundreds of near-death survivors who expressed pleasant experiences in which they left their body, viewed themselves from above, and passed down a tunnel toward a light that helped them to evaluate their life to then decide to return back to life instead of a peaceful death. These experiences reportedly left the individuals with reduced fear of death and focused on less materialistic but more life-fulfilling needs. Before the seminal study of Moody, there had been similar descriptions of such experiences in the medical and psychiatric literature, coming from many different cultures (Druss and Kornfeld, 1967; Dobson et al., 1971; MacMillan and Brown, 1971; Dlin et al., 1974; Barrett, 1986), including experiences from children (Morse et al., 1986; Morse and Perry, 1991). Up until today several aspects of these experiences and descriptions remain a matter of debate: Are these experiences survival after death or did the individuals not actually die and the experiences were part of life? If there is life after death, these experiences may provide clues but they cannot be definitive evidence that there is. On the other hand, it seems unlikely that these experiences are invented or accidental given their similarity across so many different ages and cultures. Why are these features often reported so similar? We aim to shed light on this subject by discussing the following questions: What are the neurophysiological changes in the brain that occur during these experiences and what are their anatomical correlates? What is the influence of drugs and metabolic factors involved? We review these aspects in the context of deciphering the last moments of life and attempt to provide insights into the prevailing questions of when conscious perception of life ends and ultimately when life ends.

### Clinical determination of death

In August 1968, a committee at Harvard Medical School published a landmark article to redefine the criteria on irreversible coma and death (Beecher, 1968). In addition to the traditional way, i.e., loss of heart function, the committee suggested to include the loss of neurological function, i.e., brain death. The report provided a foundation for the eventual adoption of legislation that established brain death as legal death in all 50 states of the United States. To achieve uniformity across state lines and alignment of the law with medical practice, the President’s Commission for the Study of Ethical Problems in Medicine and Biomedical and Behavior Research recommended state legislators adopt the Uniform Determination of Death Act (UDDA; United States. President’s Commission for the Study of Ethical Problems in Medicine and Biomedical and Behavioral Research, 1981). The clinical criteria for death include assessments of brain and heart function. According to the standards published by the American Academy of Neurology in 1995, brain death necessitates coma, absence of brainstem reflexes, and apnea (Wijdicks, 2001; Walter, 2020). Circulatory death is generally defined as an irreversible cessation of circulatory and respiratory function and ceasing circulation and oxygenation (Neyrinck et al., 2013). Professional organizations and consortiums have sought to formalize guidelines for brain death. The revised parameters of the American Academy of Neurology included five primary recommendations (Wijdicks et al., 2010): (1) criteria for determination of brain death from the initial guidelines in 1995 have not been invalidated by reports of patients who experienced neurologic recovery, (2) inadequate evidence to determine the minimal observation period necessary to ensure neurologic functions have irreversibly stopped, (3) complex-spontaneous motor movements and false-positive triggering of the ventilator may occur following brain death, (4) inadequate evidence to compare the safety of techniques utilized for apnea testing, and (5) inadequate evidence to establish whether newer ancillary tests may confirm cessation of brain function in the entire brain (Wijdicks et al., 2010). The World Brain Death Project sought to provide a consensus for minimum clinical criteria for brain death and published its report in 2020 (Greer et al., 2020), where it synthesized eight criteria for brain death: (1) no evidence of arousal or awareness to maximal external stimulation; (2) pupils fixed in a midsize position and unreactive to light; (3) absent corneal, oculocephalic, and oculovestibular reflexes; (4) absent facial movement to noxious stimuli; (5) absent gag reflex to bilateral posterior pharyngeal stimulation; (6) absent cough reflex during deep tracheal suctioning; (7) no brain-mediated motor response to noxious stimulation of the limbs; and (8) lacking spontaneous respirations during apnea test targets of pH < 7.30 and Paco2 ≥ 60 mm Hg (Greer et al., 2020). Per this report, ancillary testing with blood flow studies or electrophysiology, such as EEG with criteria over no detectable electrical activity (≥ 2 μV) over 30 min, may be considered when a detailed clinical examination cannot be completed but should not routinely be utilized (Greer et al., 2020). The authors of the World Brain Death Project indicated that sensitivity of 53–80.4% and specificity of 97% and concerns over confounding and interobserver reliability in existing studies limit the utility of EEG (Greer et al., 2020).

Attempts to employ electrophysiologic monitoring with EEG to determine death have been made but are currently not standard. Most patients meeting clinical criteria for brain death exhibit isoelectric EEGs (≤ 2 uV at a sensitivity of 2 uV/mm; Wijdicks, 1995). The more recent EEG-based guidelines of the Société de Neurophysiologie Clinique de Langue Française has indicated that electrocerebral inactivity may be confirmed with complete electrocerebral silence (< 2 uV) on a 30-min good-quality EEG, provided that the influences of hypothermia, metabolic disorders, and sedative drugs have been ruled out (Szurhaj et al., 2015). More specific criteria indicate that electrocerebral inactivity at sensitivity of 2 uV/mm with double-distance electrodes ≥ 10 cm apart from each other must occur under intense somatosensory or audiovisual stimulation for ≥ 30 min for EEG-based brain death (Lee et al., 2017).

### Cellular neurophysiological changes after cardiac arrest

Neurons are particularly vulnerable to ischemia due to their lack in energy stores. Ischemia leads to termination of aerobic metabolism with adenosine triphosphate (ATP) depletion. This results in dysfunctional energy-dependent Na^+^/K^+^ ion exchange pump action and as a consequence massive influx of sodium and water and intracellular cytotoxic edema. K^+^ efflux and membrane depolarization open voltage-gated calcium channels and an increase of intracellular calcium. The neurotransmitter glutamate is released and primarily binds to cell membrane receptors: (i) the mGlu receptor, which releases calcium stores from the endoplasmic reticulum *via* the intracellular mediator IP3; and (ii) the N-methyl-D-aspartate (NMDA) receptor, which also permits calcium influx. The increased calcium levels activate calcium-dependent lytic enzymes, including caspases, proteases, and phospholipases, which destroy the cell structure. Calcium also enters mitochondria and dysregulates the electron transport. The overall consequence is a production of reactive oxygen species (ROS), that lead to additional cell damage and energy supply failure, inducing a vicious cycle of cell death and injury (Sandroni et al., 2021). These processes following acute ischemia are also accompanied by short-term changes in gene expression and transcriptional events (Ferreira et al., 2018).

### The neurophysiology of near-death experiences and the dying brain

Just like birth to begin life, every human being experiences death. Yet we know so little about it. Some of the fundamental basic questions that are unanswered are: What do we feel when we die? When are we death? What happens in our body and brain when we die? Near-death experiences (NDEs) are perceptual experiences that include emotional, spiritual, and mystical components. National sample surveys among the general public revealed that approximately 4–8% of people experienced NDEs (Knoblauch et al., 2001; Perera et al., 2005). This special state of consciousness (Martial et al., 2020) has been collected from people all around the world across several centuries and from various cultural backgrounds. The experiences from NDE survivors are very valuable in understanding the process of dying. To better understand and categorize NDEs, the Greyson NDE scale (Table 1) was developed (Greyson, 1983) as a 16-item self-report questionnaire. The hallmarks of NDEs include a recall of life, memory flashbacks, out-of-body experiences, meditative states, and altered levels of conscious perception and awareness. An attempt to better understand the neurophysiological signature of NDEs is to identify the neural correlates of these experiences in healthy subjects. The knowledge gathered from these experiments can then be used to interpret recordings from dying patients to understand the neurophysiological signature around the time of death.

**Table 1:** Greyson near-death experience scale.

Question	Value	Meaning
Did time seem to speed up or slow down?	0	No
	1	Time seemed to go faster or slower than usual
	2	Everything seemed to be happening at once; or time stopped or lost all meaning
Were your thoughts speeded up?	0	No
	1	Faster than usual
	2	Incredibly fast
Did scenes from your past come back to you?	0	No
	1	I remembered many past events
	2	My past flashed before me, out of my control
Did you suddenly seem to understand everything?	0	No
	1	Everything about myself or others
	2	Everything about the universe
Did you have a feeling of peace or pleasantness?	0	No
	1	Relief or calmness
	2	Incredible peace or pleasantness
Did you have a feeling of joy?	0	No
	1	Happiness
	2	Incredible joy
Did you feel a sense of harmony or unity with the universe?	0	No
	1	I felt no longer in conflict with nature
	2	I felt united or one with the world
Did you see, or feel surrounded by, a brilliant light?	0	No
	1	An unusually bright light
	2	A light clearly of mystical or other-worldly origin
Were your senses more vivid than usual?	0	No
	1	More vivid than usual
	2	Incredibly more vivid
Did you seem to be aware of things going on elsewhere, as if by extrasensory perception (ESP)?	0	No
	1	Yes, but the facts have not been checked out
	2	Yes, and the facts have been checked out
Did scenes from the future come to you?	0	No
	1	Scenes from my personal future
	2	Scenes from the world’s future
Did you feel separated from your body?	0	No
	1	I lost awareness of my body
	2	I clearly left my body and existed outside it
Did you seem to enter some other, unearthly world?	0	No
	1	Some unfamiliar and strange place
	2	A clearly mystical or unearthly realm
Did you seem to encounter a mystical being or presence, or hear an unidentifiable voice?	0	No
	1	I heard a voice I could not identify
	2	I encountered a definite being, or a voice clearly of mystical or unearthly origin
Did you see deceased or religious spirits?	0	No
	1	I sensed their presence
	2	I actually saw them
Did you come to a border or point of no return?	0	No
	1	I came to a definite conscious decision to “return” to life
	2	I came to a barrier that I was not permitted to cross; or was “sent back” against my will

#### Basis of neurophysiological measurement and types of EEG recordings

**Figure 1:**
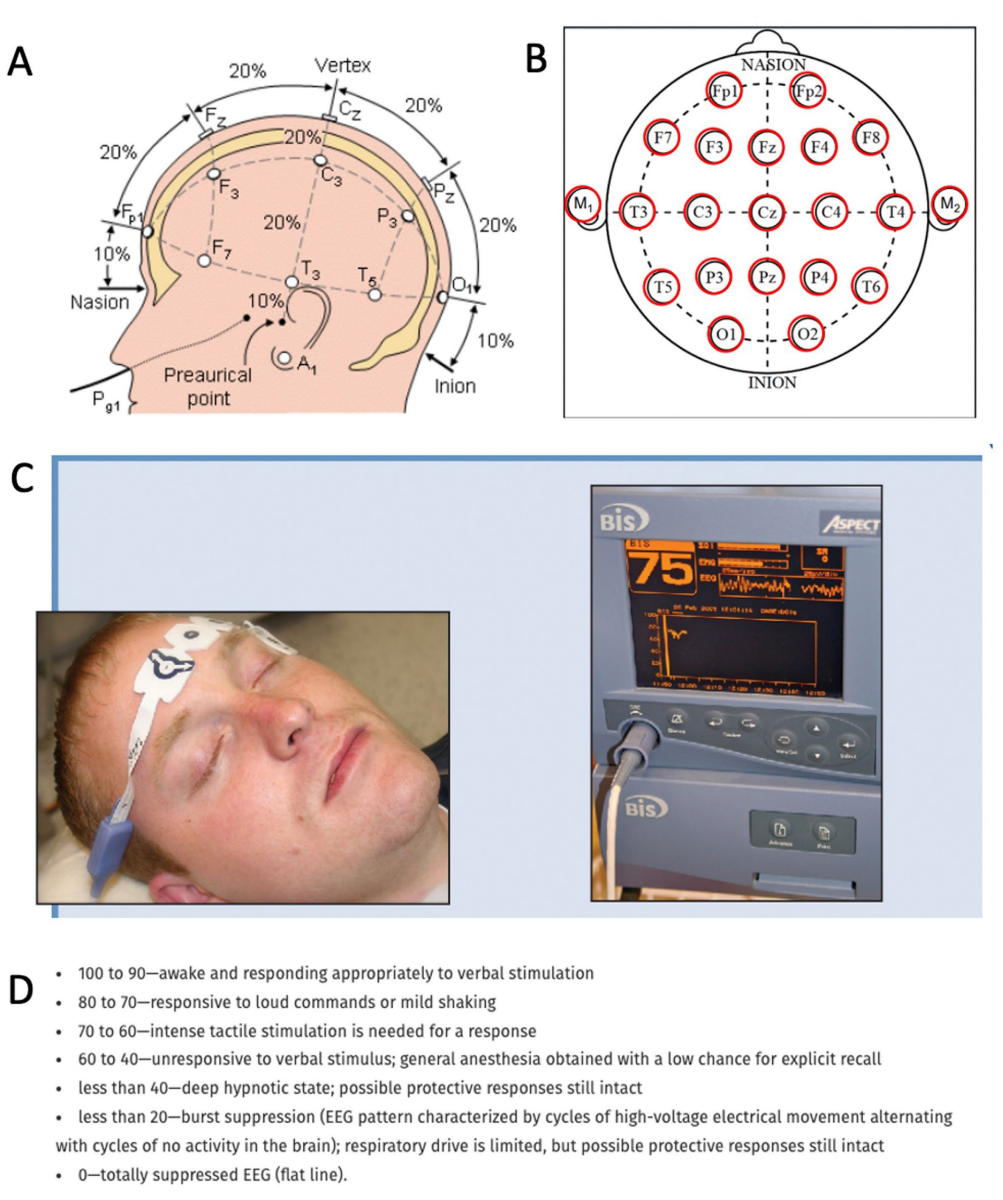
Methods to record neurophysiological activity in the dying human brain. **(A,B)** Standard 10–20 EEG lead placement and coverage of brain areas. **(C)** Placement of a bispectral index system. Recording patches are placed in the forehead to obtain EEG data and monitor enteral consciousness. Image obtained from Donaldson M, Goodchild JH. Use of bispectral index system (BIS) to Monitor Enteral Conscious (moderate) sedation during general dental procedures. Adapted with permission from Donaldson and Goodchild, Journal of the Canadian Dental Association. 2009 Dec 1; 75 (10). **(D)** Frequencies of EEG waveforms with corresponding states of consciousness.

The central nervous system relies on proper and intricate functioning of its connected assemblies and networks. As such, the activity of neurons influences other neurons through excitation or inhibition. As a result, the neuronal networks are rhythmically activated and inhibited. This rhythmicity is captured by the electroencephalogram (EEG), which reflects the rhythmic activity of extracellular field potentials, known as local field potentials (LFPs). In other words, the LFP is a neural voltage fluctuation recorded from the extracellular space, which mainly originates from postsynaptic potentials. A neural oscillation is a periodic and wave-like variation of the neural signal. The frequency of the neural oscillation is determined by various constants and network properties and spans from slow activity where the oscillation lasts several seconds to rapid activity where an oscillatory cycle completes within milliseconds. Different frequencies can occur in the brain at the same time, their waveform activity is divided in different frequencies that spans from very slow (0.01 Hz) to very fast (600 Hz; Buzsaki, 2006), whereas typical EEG in the clinical setting is contained between approximately 1 and 150 Hz. The frequency bands have been historically given different names from the Greek alphabet: delta (0.5–4 Hz), theta (4–8 Hz), alpha (8–13 Hz), beta (13–30 Hz), and gamma (30–100 Hz). Human scalp EEG can measure this activity in a non-invasive way. Each EEG electrode can capture the spatiotemporal neural network activity of the respective area where the electrode was placed. The captured signal can provide information on the phase relation of the EEG signal between two regions (phase synchronization) or the EEG power, i.e., the square of the EEG signal amplitude, within one region. Local changes in power originate from synchronized postsynaptic potentials of a large number of neurons within approximately 1 cm^2^ of cortical surface (Buzsaki, 2006). Currently, the most widely utilized tool to record neurophysiological activity in the dying brain (and perhaps the only method allowing brain activity capturing in the acute setting) is the EEG. EEG recordings can be done in two ways in the clinical setting: The first method is a full brain standard 10–20 EEG scalp recording (Figures 1A,B), which is an internationally recognized and standardized method consisting of 16 channels placed with equal inter-electrode spacing to cover all brain regions (Morley et al., 2016). Coherently, the 10–20 EEG recording provides the ability to assess oscillatory activity in all brain regions. The second method is the so-called Bispectral Index (BIS) monitor, which consists of 2 to 4 channels that are placed on the patient’s forehead (Rosow and Manberg, 2001; Donaldson and Goodchild, 2009). The sensors receive EEG signal from their respective area and send the signal to a monitor, which calculates the data as a numeric value from 0 to 100 to provide an assessment about the patient’s degree of sedation (Figures 1C,D). This tool was primarily developed to monitor the effects of anesthesia and depth of sedation. It was not aimed for detailed spatiotemporal analysis of brain neurophysiology. Because it only consists of 2–4 electrodes placed on the forehead, it can only provide information from neuronal network activity captured in that area. It does not capture oscillations outside of the frontal region of the brain. Although both methods provide information about oscillatory activity in the brain, it is important to note their spatial differences. Because the standard 10–20 EEG will capture all brain regions while the BIS monitor only reflects activity from the frontal region, the data on oscillatory activity obtained from these two methods are likely to vary.

#### Functional significance of neuronal oscillations in healthy subjects

What functional role do neuronal oscillations have for tasks and behaviors under normal daily life conditions? Neural oscillations provide a temporal frame for information processing of perception, consciousness, and memory during waking, dreaming and meditation (Llinás and Paré, 1991; Llinas and Ribary, 1993; Llinás et al., 1998; Lutz et al., 2004; Beauregard and Paquette, 2008; Fries, 2009). Activity at the alpha band (8–12 Hz) was discovered first by Hans Berger, as it has a very prominent activity peak visible on the EEG when the participant closes the eyes. Due to that relationship with a state where the eyes are closed, the alpha rhythm has been seen as a correlate of the idling state (Palva and Palva, 2007). The modern view gives the alpha band more active functions and relates it primarily to the inhibition of irrelevant signals (Klimesch et al., 2007; Palva and Palva, 2007; Klimesch, 2012). A similar inhibitory function has also been suggested for delta band activity, which is thought to suppress networks that are not essential for task accomplishment (Harmony, 2013). Beta oscillations have been widely linked with movement, in particular during stable postures or akinesis but also in perceptual and cognitive processes (Schmidt et al., 2019). Theta band activity (4–8 Hz) has been known to exist in the hippocampus of different species since the middle of the 20th century (Green and Arduini, 1954) and has been historically related to navigation (Buzsaki, 2006) but also for memory processing (Herweg et al., 2020) and memory recall, especially in verbal and spatial memory and during meditation (Siapas et al., 2005; Kahana, 2006; Vicente et al., 2022). Finally, high-frequency gamma band activity (> 40 Hz) has been attributed as a correlate of attention and awareness (Fries, 2005; Gregoriou et al., 2009), out-of-body experiences (Jensen et al., 2015; Zeev-Wolf et al., 2017) meditative states (Lee et al., 2018), and conscious perception (Singer and Gray, 1995; Singer, 2001; Melloni et al., 2007). The latter emerged from the earlier binding-by-synchrony hypothesis stating that for objects to be perceived coherently, synchrony has to be established between the different neural populations coding for the different features of the object (Singer and Gray, 1995). Evidence supporting an underlying role for gamma oscillations for conscious perception was provided by an early study: When the participants consciously perceived a face, long-distance gamma synchrony was evident in the EEG (Rodriguez et al., 1999). In another study, gamma activity was directly correlated with conscious perception as in those trials where the participants consciously perceived the stimulus, synchronization was observed in the gamma range (Melloni et al., 2007). On the other hand, more recent evidence has questioned the exact role of gamma-activity in conscious experience (Aru et al., 2012a; Pitts et al., 2014; Koch et al., 2016), emphasizing the fact that conscious experience can change without alterations in gamma activity (Aru et al., 2012a). This suggests that the gamma signal is more related to consequences of conscious processing than consciousness itself (Aru et al., 2012b; Pitts et al., 2014).

Gamma oscillations have not only been observed during awake consciousness but also during sleep. Sleep usually begins with light slow-wave sleep (SWS), characterized by large slow waves (0.5–4 Hz) that are distinct from waking rhythms. Light SWS is followed by a deeper SWS, back to shallow SWS and then rapid eye movement (REM) sleep, in which LFP recordings look similar to the waking state with small-amplitude gamma waves in the neocortex and theta-nested gamma oscillations with the hippocampus (Watson and Buzsáki, 2015). A number of studies, however, have also reported gamma activity during SWS and anesthesia, when consciousness is believed to be reduced (Steriade, 2006; Chauvette et al., 2011; Valderrama et al., 2012), contrasting the idea that gamma oscillations could represent a direct correlate of consciousness. Within SWS, there is a depolarizing (ON) phase, which shares similar electrophysiological properties with wake state activity (Destexhe et al., 2007; Constantinople and Bruno, 2011; REFS). To this end, studies report gamma activity to be nested within the depolarizing ON phase of SWS (Steriade et al., 1993; Timofeev and Steriade, 1996; Steriade and Paré, 2007; Hwang et al., 2013), arguing that gamma activity has similar properties to those during awake. Although the presence of gamma oscillations during SWS and anesthesia raises caution in the relationship between conscious processes and gamma activity, the current literature provides evidence that gamma-band activity is involved in conscious perception (Melloni et al., 2007; Aru et al., 2012a; Pitts et al., 2014). This high-frequency band also plays an important role in memory processing (Fell and Axmacher, 2011). Enhanced gamma activity was observed during viewing of pictorial stimuli and attributed to successful memory encoding and retrieval (Jutras et al., 2009). Correct odor identification in a sampling task was accompanied by increased coherence of hippocampal theta and gamma field activation (Rangel et al., 2016). Cortical gamma oscillations are also enhanced during behaviorally important stimuli or incidental stimuli that are recognized from past experience (Brunet et al., 2014). Another study revealed a distinct pattern of gamma oscillations between hippocampus and cortex to distinguish true from false memories (Sederberg et al., 2007a). In one series of studies, intracranial electrodes were implanted in epilepsy patients while they were asked to study a list of individually presented words and recall them later (Sederberg et al., 2003, 2007b). It was observed that gamma oscillations in the hippocampus and the left temporal and frontal cortices predicted successful encoding of new memories. Phase coupling of neurons to faster theta and gamma oscillations was associated with co-firing at short latencies in the medial temporal lobe and occurred during successful episodic memory formation (Roux et al., 2022). During memory retrieval, gamma activity measured with MEG was stronger for recognized items (Osipova et al., 2006). Using direct intracranial recordings, investigators researched whether the posteromedial cortex, a hub for self-referential processing, is relevant for the retrieval of self-related episodic memories (Foster et al., 2012). It was observed that the recall was associated with increases in broadband gamma signals. Working memory, i.e., the ability to keep information in mind for short periods of time, relies on attention and the ability to ignore tasks irrelevant to the subject. While alpha-band activity is known for the latter, gamma oscillations were significantly increased in the parietal lobe during working memory recall (Thompson et al., 2021). This interplay between the gamma-and alpha band also seems to be critical for a phenomenon that is often described by near-death survivors: Out-of-body experiences.

Bodily self-consciousness depends on self-identification with the body, self-location, and the first-person perspective known to be a multisensory process (Blanke, 2012). Many of the studies on bodily self-consciousness have examined out-of-body experiences, in which the individual is awake and views their body from a location outside of it (Blanke et al., 2004). In a study utilizing conflicting visual-somatosensory input of a virtual body in front of them, participants indicated that the virtual body was theirs and mistakenly localized themselves to the virtual body (Lenggenhager et al., 2007). Where in the brain are out-of-body experiences localized and which neurophysiological dynamics are triggered when we experience them? Stimulation of the right angular gyrus in a patient undergoing evaluation for epilepsy treatment promoted repeated out-of-body experiences along with illusory transformations of the arms and legs and whole-body displacements (Blanke et al., 2002), whereas damage to the right posterior superior temporal gyrus leads to improper self-location and first-person perspective (Blanke, 2012). Out-of-body experiences and autoscopy occur due to a failure to integrate proprioceptive, tactile, and visual information regarding one’s body, described as disintegration in personal space, and vestibular dysfunction leading to discordance between personal and extrapersonal space, labeled as disintegration between vestibular and visual space (Blanke et al., 2004). Investigators have extended findings from out-of-body and heautoscopy experiments to determine that bodily agency plays a causal role, but is not required, for phenomenal selfhood (Blanke and Metzinger, 2009). The temporo-parietal junction (TPJ) has been identified as the anatomical substrate for the spatial unity of self and body (Blanke and Arzy, 2005). Underscoring the function of alpha-band oscillations as an interceptor of irrelevant signals, alpha activity is increased at the TPJ during out-of-body experiences to inhibit the integration between body and space while enhanced gamma activity is thought to foster the autoscopic phenomenon through reticulogeniculo-cortical activation (Blanke, 2004; Blanke et al., 2005; Blanke and Arzy, 2005; Blanke and Thut, 2007; Ravinder et al., 2016; Zeev-Wolf et al., 2017; Milne et al., 2019).

In summary, the experiences that are described by near-death survivors, including memory recall, conscious perception, or out-of-body experiences are linked with increased activity of specific oscillatory bands in healthy subjects. Hence, the question arises whether these oscillatory bands are present during near-death experiences and in the dying brain.

#### Functional role of neuronal oscillations and temporal dynamics in the dying brain

Memory recall is a hallmark of NDEs. Recurrent descriptions of near-death survivors include the experience of autobiographical memories; NDEs themselves are also remembered well and subsequently described in much detail (Martial et al., 2020). To study the recollection of NDEs, researchers invited a group of individuals who had experienced an NDE and instructed them to recall the NDE (Martial et al., 2019). Individuals were able to recreate NDE-like features during which the experience was associated with increased alpha-band activity (Martial et al., 2019). Memory recall may have distinct oscillatory patterns during NDE and in healthy subjects. Memories were linked with increased theta and delta oscillations in 10 participants with NDEs, whereas recall of life memories in 10 control subjects without NDEs were linked with enhanced alpha- and gamma-band activity (Palmieri et al., 2014). The study suggested that NDE memories cannot be considered equivalent to imagined memories but are rather stored as episodic memories of events in a peculiar state of consciousness. High-frequency gamma oscillations have received particular interest in the investigation of the dying brain. Early rodent studies observed neocortical gamma bursts shortly after decapitation (Vanderwolf et al., 1988) and induced ischemia (Freund et al., 1989). Subsequent studies confirmed the presence of EEG spikes after decapitation but with variable time spans from including an increase in EEG frequency in the first 15 s after decapitation, which was followed by a high-amplitude wave that appeared 50–80 s after decapitation, known as the “wave of death” (WoD; Rijn et al., 2011; Kongara et al., 2014). In a landmark paper, continuous EEG was recorded to investigate neurophysiological changes in the dying brain of rats after induced cardiac arrest. An increase of gamma oscillations was noted in all brain regions 30 s after cardiac arrest, which preceded isoelectricity in the EEG. The gamma surge was accompanied by increased anterior–posterior connectivity and phase-coupling with theta-and alpha-bands. The authors concluded that the mammalian brain is capable of generating neural correlates of active conscious processing at the near-death state (Borjigin et al., 2013). Surprisingly, this coordinated activity outlasted termination of heart function. The latter addresses the question on which organ dies first, the heart or the brain and with that the question on when we actually really die.

The classic view regarded electrocardiogram (ECG) activity as the key component to determine death and considered brain function to cease with blood flow cessation, which is reflected in a flat-lined EEG. EEG has been applied to patients with different pathologies, including cardiac arrest and cardiac death. Early epileptiform EEG occurs in one-third of patients following cardiac arrest and is associated with a poor prognosis (Westhall et al., 2016; Rossetti et al., 2017; Faro et al., 2019; Barbella et al., 2020). The prognostic value of early epileptiform EEG, defined as < 72 h after cardiac arrest, can be predicted with timing, continuity, reactivity, amplitude, and discharge variables (Barbella et al., 2020). In this study, a composite score, in which 1 point was assigned to each of the following elements portending good prognosis—no epileptiform activity, continuity ≥ 50%, reactive background on 1st EEG reactive background, formal background amplitude, stimulus-induced rhythmic periodic or ictal discharge—was determined. An increasing composite score correlated with neuronal damage. Adding a series of quantitative EEG features, including power spectral density, local coherence, and permutation entropy measured at days 4–6 after cardiac arrest to previously validated metrics of clinical recovery mildly increases prediction accuracy for the recovery of consciousness following cardiac arrest (Bauerschmidt et al., 2021). The notion of the brain seizing activity prior to the heart was backed by studies that provide evidence of a fairly rapid flat-lined EEG that occurs prior to ECG termination. In a case series of four patients, frontal region EEG inactivity through BIS electrodes occurred prior to ECG and arterial blood pressure inactivity in 3 patients, while the fourth patient exhibited irregular single delta bursts for more than 10 min after ECG termination (Norton et al., 2017). A similar observation was made when whole brain EEG was recorded in 19 patients, of which only 2 demonstrated EEG activity after the last QRS complex in the ECG (Matory et al., 2021). On the contrary, the matter seems to be more complex when recordings are not restricted to certain brain regions or are obtained from different depths. This may have been noted early on when in 1969 a survey of members of the American Electroencephalographic Society recognized the temporal importance by suggesting the absence of cerebral activity on EEG for *30 min* to determine brain death (Silverman et al., 1969), indicating that brain activity may not terminate abruptly over a short time but rather gradually. Subsequent studies investigated EEG patterns of death in more detail: An early study reported three categories of EEG activity after brain death for a mean duration of 36.6 h: low-voltage (4–20 uV) theta or beta activity, sleep-like activity (mixture of synchronous 30–40 uV theta and delta activity and 60–80 uV/10–12 Hz spindle-like potentials), and alpha-like activity (monotonous, anteriorly predominant 20–40 uV/9–12 Hz activity; Grigg et al., 1987). Post cardiac arrest EEG activity obtained from frontal region BIS electrodes was associated with good prognosis to recover awareness, whereas generalized suppression or epileptic discharge activity on the EEG correlated with poor recovery (Thenayan et al., 2010). Frontal EEG measurements revealed a decline in BIS activity immediately after loss of blood pressure through the arterial line but the frontal EEG decline was followed by a transient spike that approximated levels normally seen with consciousness (Chawla et al., 2009). The same group investigated the presence of so-called end-of-life electrical surges (ELES) in 35 patients. No ELES were found in 7 patients that were declared brain death, whereas the surges were identified in 13 of the remaining 28 patients (Chawla et al., 2017). A large prospective trial included a subset of patients who recalled memories, events and cognitive themes after cardiac arrest, indicating that the brain is functionally active after cardiac cessation (Parnia et al., 2014). Similar to the observation in rodents by Borjigin et al. (2013), a recent case report recording whole brain EEG from the dying human brain revealed an increase of relative gamma oscillations 30 s post cardiac arrest. In the report from an acutely deteriorating 87-year-old patient, a surge of relative gamma power and reduction of theta rhythms was identified after suppression of neuronal activity in both hemispheres. After cardiac arrest, the relative amount of gamma power increased, while a reduction of delta, beta, alpha, and absolute gamma waves was seen. Cross-frequency coupling revealed strong modulation of the low-and broad gamma power by the alpha band (Vicente et al., 2022). A study from an Italian group recorded cortical neuron activity of macaque monkeys with a multi-electrode miniaturized array. A strong reduction of local field potentials (LFPs) and multi-unit activity (MUA) was observed after cardiac arrest (CA), which was reversed 20 min post-CA with re-emerging LFP bursts accompanied by supra- and sub-threshold MUA modulations that resulted in LFP-MUA coupling. This activity lasted for up to 2 h post cardiac arrest (Pani et al., 2018). Approximately 30,000 patients in the United States are in a so-called vegetative state and considered “awake but not aware” (The Multi-Society Task Force on PVS, 1994). Conscious perception during this state remains a largely unexplored field of medicine. Research indicates region-specific conservation of neuronal function and suggests that the brainstem is more protected than other brain regions. After simulated ischemia, neurons of the hippocampus, neocortex, striatum, thalamus and cerebellar cortex died within 10 min of ischemic stress while brainstem neurons survived under identical conditions (Brisson et al., 2014). The authors suggested the increased resilience of the brainstem to account for keeping the patient “awake,” while higher cortical centers result in the patient not being “aware.” It is important to note that the clinical diagnostic tools to assess neurophysiological function such as EEG can assess cortical activity but are not designed to measure deeper regions such as the brainstem. Therefore, although the EEG demonstrates a flat line, the activity of the brainstem can remain unknown to the clinician. Charpier et al. used an intricately designed experimental setting to investigate the functional role of the isoelectric EEG. The authors demonstrated reproducible sensory responses in humans that were evoked on the isoelectric EEG, including visually evoked potentials. Searching for intracellular correlates of this observation, the authors investigated intracellular changes of cortical and subcortical neurons during isoelectricity in rodents. Although hippocampal and thalamocortical neurons were silent, they were able to fire action potentials when depolarizing current was injected. Like in humans, during EEG isoelectricity, somatosensory-evoked potentials were also induced in rats, which was accompanied by neocortical pyramidal cell responses. The authors concluded that neurons and synapses are not terminally non-functional during an isoelectric EEG but rather dormant to conserve neuronal excitability and functioning (Altwegg-Boussac et al., 2017). Although these results were so far only captured at a microscale through intraparenchymal recordings and remain unidentified on global EEG measurements, they open the discussion on their functional relevance. Are we truly death at the time of EEG silence? What is the time window of resuscitation? Can neuromodulation reverse global ischemia within that time window? What is the timing for organ donation?

**Figure 2:**
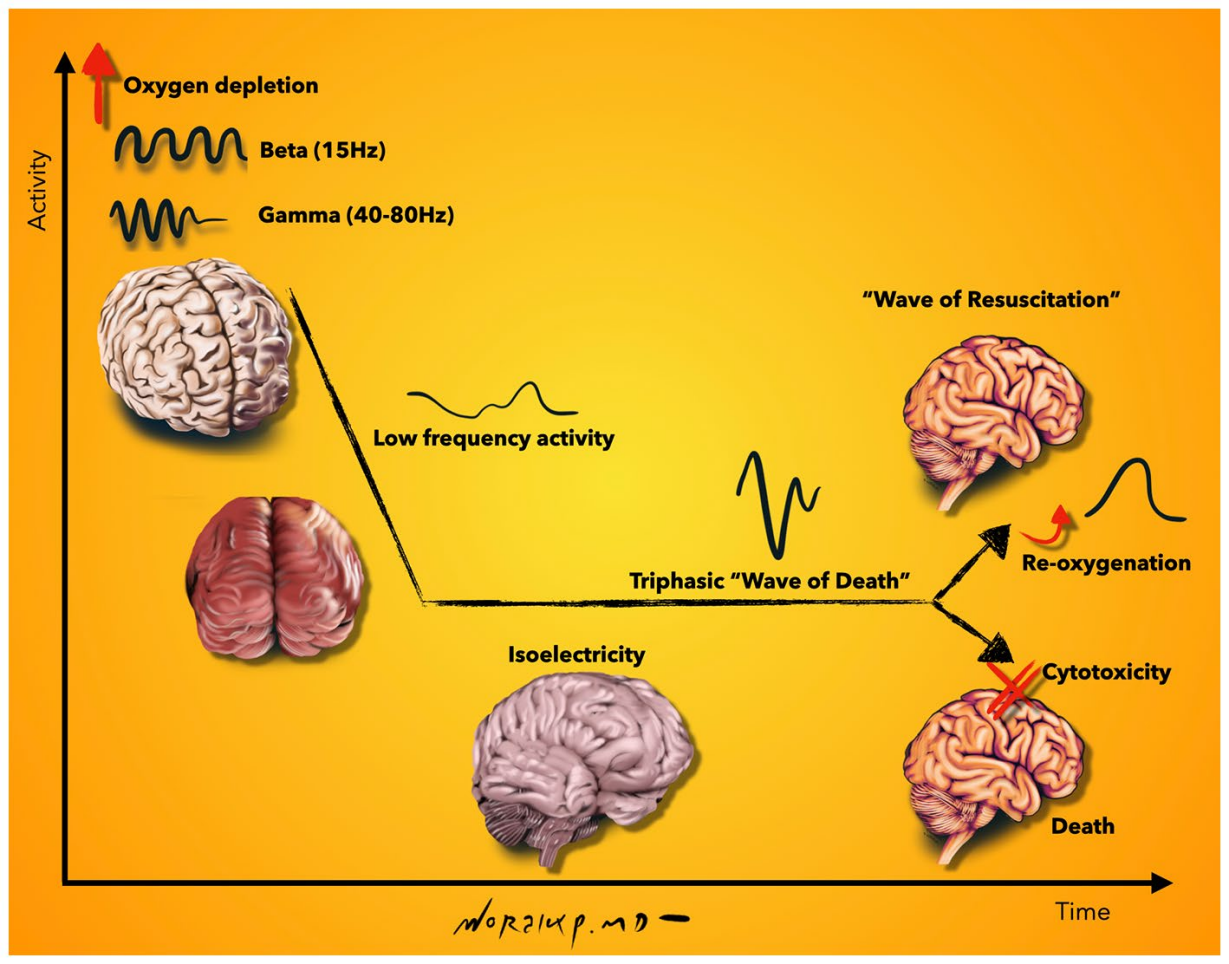
Schematic representation of neurophysiological activity during the final moments of life. Beta and gamma oscillations are increased shortly after oxygen depletion, which is followed by low frequency activity and isoelectricity. During isoelectricity, cortical neurons deploy membrane potential depolarizations, which are reflected on the EEG as a high-amplitude, sharply contoured triphasic wave, called the “wave of death.” Although all neurophysiological measurements are in line with a dead brain at this stage, a gradual repolarization can be initiated upon re-oxygenation of the brain; seen on EEG as the “wave of resuscitation.” If no re-oxygenation is supplied, loss of cell properties continue and result in cytotoxicity and death. Illustration based on findings described in Schramm et al. (2020). Illustration by Woralux Phusoongnern.

One of the most detailed neurophysiological investigations of the dying and subsequently resuscitated brain was accomplished in an elaborate study with simultaneous electrocorticographic (ECoG) and intracellular recordings from neocortical cells: A rodent model was used to first induce death by anoxia and subsequently reanimate the animal. The authors found an increase in beta and gamma oscillations in the early stage after oxygen deprivation. This occurred together with rhythmic membrane depolarizations and regular firing in pyramidal neurons. This observation was followed by low-frequency activities which further declined toward isoelectricity. During isoelectricity, cortical neurons exhibited marked membrane potential depolarizations, which were reflected in the ECoG as a large amplitude triphasic wave, the notorious wave of death. Together with anoxic depolarization, a block of action potentials and loss of cell properties were registered. Despite the fact that all neurophysiological measurements were in line with a dead brain, the functional state was reversed if brain re-oxygenation was restored within 2–3.5 min. This resulted in a gradual repolarization of neocortical neurons, initiated a second wave, which was termed as the “wave of resuscitation” and eventually re-established the pre-anoxic synaptic and firing activity (Schramm et al., 2020). Beyond rodents, the timing of recovery was also investigated in the large mammalian brain. In an elaborately designed study, scientists were able to restore molecular and cellular functions of the pig brain 4 h after electrocorticographic silence (Vrselja et al., 2019). These studies reveal interesting facts. There are different chronological phases of the dying brain (Figure 2): (i) An early surge of oscillatory activity, which is accompanied by rhythmic membrane depolarizations and regular neuronal firing, (ii) isoelectricity with massive membrane depolarizations (wave of death), block of action potentials and loss of integrative cell properties, and (iii) the ability to recover after loss of integrative cell properties (Vrselja et al., 2019). This example illustrates how difficult it is to determine the time of death electrophysiologically. While the clinical criteria of death include electrocorticographic silence > 30 min, the findings in this study demonstrate recovery after 4 h of electrocorticographic silence. While the EEG may show silence, intracellular recordings may not but they are not feasible to obtain in the clinical setting. Further clarification of these phases not only gives important clues to the temporal dynamics of dying but is also important for neuronal recovery after central nervous system injury, e.g., after stroke or traumatic brain injury. It is also important to determine whether and how long the observed neuronal activity is functionally relevant and to which degree the brain is consciously active during these phases.

### Consciousness in near-death experiences and the dying brain

Studies have investigated NDEs as a proxy to investigate neurophysiological activity in the dying brain. The incidence of conscious perception and memory within NDEs varies between articles based on study design (Greyson, 1986, 2003a,b; Parnia et al., 2001, 2014; Schwaninger et al., 2002; Van Lommel et al., 2017). Perhaps the most comprehensive work on consciousness and awareness during the transition to death has been the prospective AWARE study, which examined the experiences of hundreds of patients with cardiac arrest who had biologically crossed over the threshold of death before being resuscitated. The authors reported that 46% of cardiac arrest survivors experienced memories, 9% NDEs, and 2% awareness with recall of seeing or hearing events pertaining to their resuscitation (Parnia et al., 2014). This study did not explicitly exclude cases in which patients’ memories were based on retrospective imaginative reconstruction built up from memories, expectations about the world, or prior knowledge. NDEs have been traditionally thought to result from a disturbance of brain function during the dying process or a psychological response to the perceived threat of death (Owens et al., 1990; Blackmore, 1996; Greyson, 2000). They appear to occur when cerebral function is absent or impaired but are different from metabolic or physiological changes during hallucinations because they occur in non-functioning cortex (Parnia and Fenwick, 2002). Paradoxically, NDEs indicate an unexpected increase in awareness, attention, and consciousness (Parnia and Fenwick, 2002). NDE memories contain more characteristics than real event and coma memories (Thonnard et al., 2013) but are similar to real memories in terms of detail richness, self-referential information, and emotional information (Palmieri et al., 2014). Participants who describe more intense NDEs as assessed using the Greyson NDE scale also report more phenomenological memory characteristics of NDEs (Martial et al., 2017). NDE intensity is associated with sensory details, personal importance, and reactivation frequency (Martial et al., 2017). Some have argued that a framework with axes of awareness, wakefulness, and connectedness is necessary to conceptualize NDEs as they are thought as an internal awareness that is experienced in conditions of unresponsiveness and comprise an event of disconnected consciousness (Martial et al., 2020). The theories and hypotheses around the link of consciousness and NDEs are controversial and difficult to prove given the difficulty of timely and accurately recording activity in the dying brain. Moreover, they are influenced by a variety of physiological, autonomic, iatrogenic, and pathologic factors.

No patients experiencing brain death have returned back to life (Wijdicks et al., 2011; Wahlster et al., 2015; Varelas et al., 2021). Studies examining brain death mimics indicate decreased arousal but do not examine perception or memory formation (Ostermann et al., 2000; Vargas et al., 2000; John et al., 2008; Peter et al., 2008; Bernard et al., 2010; Sullivan et al., 2012; Ravikumar et al., 2016; Stranges et al., 2018; Libonati et al., 2021; Pearson et al., 2021). One study on Guillain-Barre mimicking brain death determined that EEG showed an alpha rhythm and normal evoked potentials (Vargas et al., 2000). Moreover, Martial et al. (2022) argue that these do not consider the element of the brain death protocol that requires well-documented, irreversible, fatal brain damage (Wijdicks, 2015), a provision reinforced by the more recent World Brain Death project criteria (Greer et al., 2020). As a result, conclusions from NDEs can only be utilized as a, likely imperfect, proxy for consciousness in the dying brain. Extending findings from NDE examinations, it is possible that individuals who are dying may experience hyperawareness, hyperattention, and an enhanced state of consciousness, with a predominant recall of more salient, emotional, and self-referential memories.

### Data variability and possible sources of contamination

#### Region, duration, and depth of recordings

The studies described above reveal that there is variability among them. How can this be explained? The articles discussed in this review reveal that different results can be obtained depending on *where*, *how long* and *which cells* are being recorded. Different brain regions may die at different times and not all at once. As such, recordings through BIS electrodes capturing only frontal regions may show different results than whole-brain recordings capturing all brain regions. Even comparing the frontal leads of a standard EEG recording can vary from the signals captured through BIS electrodes, likely because the algorithms through which these systems capture brain activity vary. The sequence of the different neurophysiological phases illustrated in Figure 2 discloses that one may conclude a flat-lined EEG if the EEG is stopped during the isoelectric phase and one may find a different result if the EEG is continued further until the second depolarization is noted. The studies that analyzed intracellular and intraparenchymal recordings also revealed different time stamps than EEG recordings resulting in large temporal variability within which the neurophysiological brain activity is captured. The studies mentioned above report a time window of cerebral activity spanning from EEG cessation prior to ECG termination up until cortical neuronal activity lasting up to 2 h post cardiac arrest.

#### Model of investigation

Studies have utilized different models to study death. These include decapitation, cardiac arrest, cervical dislocation, anoxia, potassium chloride injection, CO2 inhalation, etc. The data recorded are likely to vary between these models. Decapitation may show a different pattern than cervical dislocation, which may again be different from anoxia or cardiac arrest. Therefore, the conclusion obtained from the respective study could be compared to another study using the same model and identical recording techniques but is likely to show variability when different models are compared among each other. Moreover, a physiological assessment of the dying brain can only be obtained from animal studies as healthy subjects are not going to appear in hospitals to volunteer for analysis of their dying brain. By default, a patient in the hospital has a pathological diagnosis which brings the patient to the physician. Thus, human studies are always confounded by the underlying pathologies of the respective patient. These pathologies are likely to affect the level of consciousness and general condition of the patient, which will likely result in varying results when, e.g., consciousness during death is examined. For example, the outcomes from a traumatic brain injured patient are likely to differ from a patient without trauma. These, in turn, are likely to differ from a patient who underwent palliative care and receives comforting medications and measures but no nutrition.

#### Contamination of data

The data recorded are the foundation of any study. Analysis and conclusions are made based on the purity of the data. For example, when investigating gamma oscillations in the dying brain, the source of oscillations has to be carefully evaluated and confounding variables discussed and considered. The contaminating sources of high-frequency brain activity include muscle artifacts (Muthukumaraswamy, 2013; Ulloa, 2022), tonic pain (Schulz et al., 2015), traumatic brain injury, hematomas, swelling, seizures, anesthesia, dissociated drugs, antiepileptic drugs, asphyxia, and hypercapnia to name a few (Vicente et al., 2022). We can impossibly exclude all of these contaminating factors from datasets, especially in human subjects (animal experiments permit more controlled environments). Variability between studies will be inevitable. Similarities between studies may be an effective way to draw conclusions.

In consideration to these aspects, it is critical not to shuffle the available data into one pool but carefully separate the data based on how it was obtained to learn in the best possible way from each available piece. This is of particular importance since data in this field of research is very valuable due to the fact that the nature and timing of death is unpredictable, which impedes detailed investigations and data collection. Another approach would be an effort to perform studies under standardized guidelines for data collection that are established by participating scientists to minimize variability.

## Conclusion

The science of death has attempted to make the mystery of death less mysterious and more understandable. Detailed animal studies under controlled environments are a compelling way to investigate the underlying neurophysiological changes in the dying brain. Human studies are more difficult to conduct due to the confounding factors, varying pathologies, timing in capturing brain activity, and ethical considerations. Descriptions from near-death survivors may be our only gateway to understand what death may look like. Understanding the neurophysiological underpinnings of these descriptions in healthy subjects and correlating them with data obtained from the dying brain could be our only gateway to deciphering the neurophysiology of death. In observing these phenomena, there is a natural desire to speculate on the nature of death and on implications for spiritual questions of death and afterlife. We may never find direct evidence to correlate subjectively reported near-death experiences with the neurophysiological changes in a dying human brain because by default we cannot ask the dying patient whether they experienced a memory recall while they died. We will likely always be restricted to correlative findings linking knowledge from prior studies and trying to position new findings into this framework. These findings are our only corridor to communicate with the dying brain and attempt to receive responses that help us understand what happens in the brain when we die.

## Author contributions

NS and JA wrote the manuscript, summarized literature and prepared tables. RV wrote and revised the manuscript. AZ conceptualized and designed the article, formulated hypotheses, and wrote and revised the manuscript. All authors contributed to the article and approved the submitted version.

## Funding

AZ was supported by the Henan Provincial People’s Hospital Outstanding Talents Founding Grant Project. JA was supported by the European Social Fund through the “ICT programme” measure and the Estonian Research Council grant PSG728. AZ was supported by the Senior Specialist Foreign Expert Project of Department of Science and Technology of Henan Province, Grant Number: G2019126006.

## Conflict of interest

The authors declare that the research was conducted in the absence of any commercial or financial relationships that could be construed as a potential conflict of interest.

## Publisher’s note

All claims expressed in this article are solely those of the authors and do not necessarily represent those of their affiliated organizations, or those of the publisher, the editors and the reviewers. Any product that may be evaluated in this article, or claim that may be made by its manufacturer, is not guaranteed or endorsed by the publisher.
